# Polygenic scores: prediction versus explanation

**DOI:** 10.1038/s41380-021-01348-y

**Published:** 2021-10-22

**Authors:** Robert Plomin, Sophie von Stumm

**Affiliations:** 1 Social, Genetic & Developmental Psychiatry Centre, Institute of Psychiatry, Psychology & Neuroscience, King’s College London, London, UK; 2 Department of Education, University of York, York, UK

**Keywords:** Psychology, Genetics

## Abstract

During the past decade, polygenic scores have become a fast-growing area of research in the behavioural sciences. The ability to directly assess people’s genetic propensities has transformed research by making it possible to add genetic predictors of traits to any study. The value of polygenic scores in the behavioural sciences rests on using inherited DNA differences to predict, from birth, common disorders and complex traits in unrelated individuals in the population. This predictive power of polygenic scores does not require knowing anything about the processes that lie between genes and behaviour. It also does not mandate disentangling the extent to which the prediction is due to assortative mating, genotype–environment correlation, or even population stratification. Although bottom-up explanation from genes to brain to behaviour will remain the long-term goal of the behavioural sciences, prediction is also a worthy achievement because it has immediate practical utility for identifying individuals at risk and is the necessary first step towards explanation. A high priority for research must be to increase the predictive power of polygenic scores to be able to use them as an early warning system to prevent problems.

Research using polygenic scores emerged as a fast-growing area in the behavioural sciences during the past decade. Polygenic scores consist of sums of thousands of single-nucleotide polymorphisms (SNPs) each weighted by the effect size of its association with a target trait derived from genome-wide association studies [1].

In 2009, the first paper was published reporting a polygenic score that predicted up to 3% of the liability to schizophrenia in independent case–control samples [2]. Since then, 2783 articles using polygenic scores have been listed on the Web of Science (search terms ‘polygenic score’ OR ‘polygenic risk score’ OR ‘polygenic risk’). The largest field of polygenic score research is the behavioural sciences (Web of Science categories: psychiatry, neuroscience, behavioural science, psychology, psychology multidisciplinary, psychological development and psychology clinical, with overlapping publications removed), which accounts for 45% (*N* = 1271) of the total publications. Figure 1 shows the dramatic rise of these 1271 polygenic score publications and their 14,228 citations, reaching 4636 citations in 2020.

**Fig. 1:**
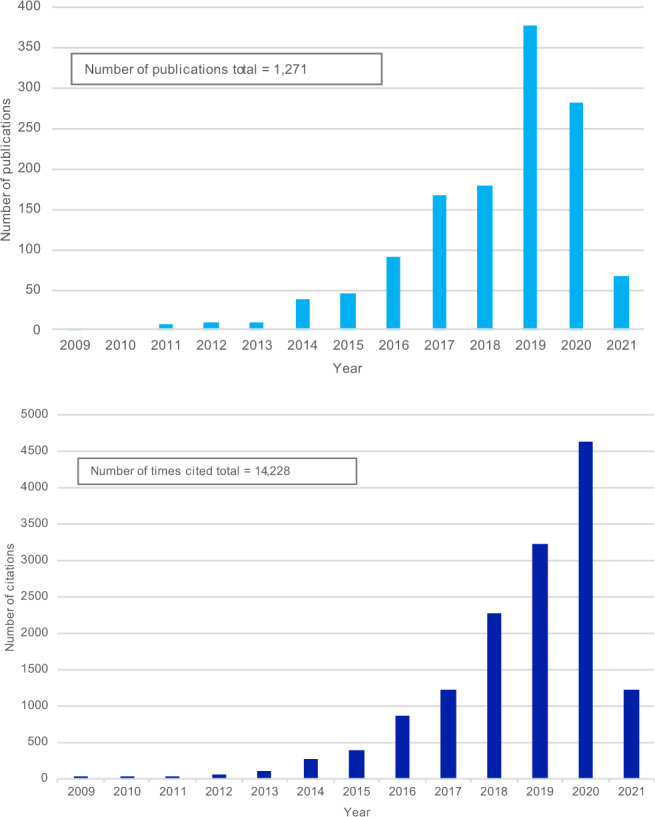
Polygenic score publications in the behavioural sciences per year since 2009. Top: number of publications. Bottom: Number of times cited. Data from Web of Science, April 2021.

## Prediction

The predictive power of polygenic scores has increased steadily during the past decade for dozens of common disorders and complex traits. For example, the polygenic score for schizophrenia, which predicted up to 3% of the liability variance in 2009, can now predict 6% [3]. Polygenic scores can predict 2% of the liability variance for major depressive disorder [4], 5% for bipolar disorder [5], 3% for neuroticism [6], 6% for attention deficit hyperactivity disorder [7] and 10% for externalising behaviours [8]. In the cognitive realm, variance predicted by polygenic scores is 7% for general cognitive ability (intelligence) [9], 11% for years of schooling (educational attainment (EA)) [10] and 15% for tested school performance at age 16 [11], which is the most predictive polygenic score in the behavioural sciences.

Explain is the word used in statistical parlance to refer to effect sizes, but the word predict is more appropriate because polygenic scores do not explain how inherited DNA differences become associated with behavioural traits. Polygenic score predictions of behavioural traits are correlations and correlations do not imply causation. Causation is a complicated concept that generally refers to mechanisms that precede effects, often identified by experimental manipulation. Here, however, we refer to explanation in the more limited sense of statistical models of nonexperimental data that attempt to infer causation from correlational data [12, 13].

The purpose of this perspective is to contrast prediction and explanation. Prediction and explanation offer different scientific perspectives, and neither is right nor wrong, just more or less useful to achieve different research goals. The goal of prediction is to account for as much variance as possible, without regard for explanation. The goal of explanation is to deduce causality, without regard for prediction [14, 15]. These perspectives can be complementary, for example, if explanatory models are validated in terms of prediction, and if knowledge of causal processes leads to better prediction.

The value of explanation without prediction is seldom questioned but we argue here that prediction without explanation is also valuable. This point is widely acknowledged in some scientific disciplines, for example, in artificial intelligence where machine learning is an increasingly popular tool for prediction that explicitly eschews explanation. However, in the behavioural sciences, evidence for prediction has often been downplayed and devalued if it was devoid of explanation. This attitude seems especially paradoxical in the context of genomic research because success in identifying DNA differences came only after the search for candidate genes selected for their possible causal connection to a trait was superseded by a hypothesis-free approach that is agnostic about the specific function of DNA variants (i.e., genome-wide association).

The predictive power of polygenic scores is groundbreaking. Predicting 10% of the variance marks an important milestone because effect sizes of this magnitude are large enough to be ‘perceptible to the naked eye of a reasonably sensitive observer’ [16]. Nonetheless, 10% of the variance is equivalent to a correlation of only 0.32, and the resulting oval-shaped scatterplot between the polygenic score and a trait indicates the probabilistic nature of polygenic score prediction at the level of an individual [17, 18]. Even so, useful predictions can be made at the extremes. For example, the lowest and highest deciles for the polygenic score for IQ yield mean IQs of 92 and 108, respectively. For the polygenic score for EA, 25% of those in the lowest decile go to university as compared to 75% of those in the highest decile [18]. Being in the top decile of polygenic scores for schizophrenia is associated with an odds ratio of 4.6 for becoming diagnosed with schizophrenia as compared to the bottom decile; this is similar to the risk that either smoking or diabetes poses for experiencing coronary artery disease [19].

Polygenic score prediction compares favourably with other predictors in the behavioural sciences, which are rarely subjected to the same harsh spotlight of effect size. For example, in contrast to polygenic scores that predict 15% of the variance in tested school performance in the UK at age 16, ratings of school quality obtained by an independent body of evaluators (Ofsted) only predict 4% of the variance in the same tests of school performance [20]. Despite its modest effect size, school quality ratings are used by parents to decide which schools their children will attend [21].

Polygenic scores will never predict complex traits with perfect precision because heritabilities are about 50% for most behavioural traits [22]. Other limitations can be surmounted, most notably, the ‘missing heritability’ gap between variance predicted by polygenic scores and twin study estimates of heritability [23]. The missing heritability gap will be narrowed with bigger and better genome-wide association studies and with whole-genome sequencing that assesses all DNA differences in the genome rather than several hundred thousand SNPs assessed in current studies [24]. The only way is up for the predictive power of polygenic scores.

The ability to directly assess people’s genetic propensities has transformed research by enhancing the power and precision of genetic research on diagnoses and dimensions, heterogeneity and co-morbidity, developmental change and continuity and gene-environment correlation and interaction [25]. Polygenic scores make it possible to add genetic predictors of behavioural traits to any research without the need for samples of twins or adoptees. Although genome-wide association studies require huge sample sizes, a polygenic score that predicts 5% of the variance only needs a sample of 120 to detect its effect with 80% power (*p* = 0.05, one-tailed).

Polygenic score predictions are correlations, and correlations do not necessarily imply causation. However, polygenic scores have a unique causal status among predictors in one important sense: correlations between polygenic scores and traits can only be interpreted in one direction causally. That is, there can be no backward causation in the sense that the brain, behaviour or the environment cannot change inherited DNA variation. The unchanging nature of inherited DNA variation from the moment of conception also makes polygenic score predictions unique in that they are just as predictive of adult traits early in life as they are in adulthood.

## Explanation

Causal models using genomic data are burgeoning [12]. Much of this work considers the extent to which assortative mating, genotype–environment (GE) correlation and population stratification contribute to polygenic score prediction. Assortative mating is an ingredient in polygenic score prediction because it increases genetic variance in a population when individuals inherit trait-relevant DNA variants from both parents that deviate in the same direction from the population mean. GE correlation can affect polygenic score prediction, for example, when the correlation between children’s polygenic scores and their school performance is mediated by their experiences at home or school. Population stratification, such as ancestral or regional differences within a population, also contributes to the total genetic variance in the population that is predicted by polygenic scores.

Quantitative genetic research that uses family, twin and adoption designs to disentangle nature and nurture provides a backdrop for genomic studies of these processes. For example, a clever combination of twin and partner data indicated that assortative mating is caused by social homogamy rather than genetic influence on choice or environmental convergence of spouses over time [26]. However, assortative mating increases genetic variance regardless of its causal mechanisms that drive assortative mating. Most twin studies ignore assortative mating and thus underestimate heritability by misattributing its variance to shared environmental influences. This is especially the case for cognitive traits, which show much greater assortative mating than personality or psychopathology [27].

Forty years of quantitative genetic research on GE correlation has revealed that most environmental measures widely used in the behavioural sciences show substantial genetic influence, about 25% heritability on average [28–30] and correlations between environmental measures and behavioural traits are substantially mediated genetically, about 50% on average [31, 32]. Three types of GE correlation have been investigated: passive, evocative and active [33]. Passive GE correlation occurs when children passively inherit environments correlated with their genetic propensities. For example, parents with high polygenic scores for EA not only transmit high EA scores to their children but also provide experiences such as tuition, aspirations and role models that foster EA-related traits in their children. Children with high EA scores might also evoke reactions from others such as teachers who enhance their school performance. Active GE correlation occurs when children select, modify or create environments correlated with their genetic propensities. For instance, children with high EA scores might select like-minded friends, extract more information from classroom instruction and read more. Passive GE correlation is limited to experiences provided by genetically related individuals, evocative GE correlation includes experiences with anyone and active GE correlation encompasses experiences with anything.

Twin studies commingle GE correlation in their estimates of heritability, but adoption designs [31] and combinations of twins and multi-generational families [34] are able to disentangle the three types of GE correlation. For example, comparing adoptive and nonadoptive families can assess passive GE correlation because it is absent in adoptive families. Results from such research point to the importance of passive GE correlation for cognitive traits [31] and evocative GE correlation for psychopathology [35]. It has been difficult to pin down active GE correlation in part because measures of the environment widely used in the behavioural sciences assess the environment that happens to us passively rather than the experiences that we actively choose and create.

Quantitative genetic research has had much less to say about population stratification. Because ancestral and regional groups are usually included in twin analyses, their effects, which are solely between-family effects, are read as shared environmental influence.

Genomic methods have created many new opportunities to investigate assortative mating [36, 37], population stratification [38, 39] and especially GE correlation [40–42]. Some genomic methods estimate the joint effect of all three mechanisms, most notably comparing polygenic score predictions between families; polygenic score predictions within families exclude the effects of assortative mating and population stratification [10, 41, 42].

All these methods indicate that assortative mating, passive GE correlation and population stratification can contribute to polygenic score predictions. The most notable finding is that they contribute much more to polygenic score predictions of cognitive traits than other behavioural domains. This seems likely to be part of the reason why polygenic scores are more predictive for cognitive traits.

## Prediction versus explanation

Assortative mating, GE correlation and population stratification are interesting in their own right, and it is also reasonable to investigate the extent to which they contribute to polygenic score predictions. However, proclaiming that these processes make polygenic scores confounded, biased or inflated as predictors confuses explanation and prediction.

From the perspective of predicting individual differences in a particular population, that population’s assortative mating, GE correlation and population stratification are legitimate sources of genetic variance for polygenic score prediction. If our goal is prediction, we would not want to ‘correct’ the polygenic score to remove genetic variance that can be ascribed to assortative mating, GE correlation or population stratification. In contrast, in causal models such as Mendelian randomisation [41], these phenomena are viewed as confounds that need to be controlled, although it is inherently difficult to infer causality from correlational data [13].

Most controversial is population stratification, which is so assumed to be a confounder that its genetic variance is removed in the first step of genome-wide association studies by covarying principal component scores for groups that differ in SNP resemblance. Polygenic scores are corrected again for group principal components in analyses of their association with a phenotype. The chopsticks example [43] illustrates the issue: in a study of the use of chopsticks, any SNP differences between Asians and non-Asians would be incorporated in a polygenic score predicting chopstick use even though culture is the explanation for the use of chopsticks. However, it could be argued from a predictive perspective that once a phenotype and a population are defined, any inherited DNA differences that predict the phenotype in that population are legitimate sources of polygenic score prediction, whether due to ancestry, geography or culture. In addition, removing genetic variance due to ancestral differences raises the question of when to stop correcting polygenic scores because, in the end, all genetic variance is ancestral. The issue of whether population stratification confounds polygenic score prediction in a particular population is separate from the ability of polygenic scores to predict in different populations [44] or the need for greater ancestral diversity in genome-wide association studies [45, 46].

The long-term goal of the behavioural sciences is to map the explanatory pathways from DNA through the brain to behaviour [47]. Yet, prediction is the necessary first step towards explanation. Polygenic scores also have immediate impact on research, are of practical utility for identifying individuals at risk and serve as an early warning system to prevent problems before they occur.

From the prediction perspective, anything that improves the predictive power of polygenic scores is welcome, such as improved methodologies for creating polygenic scores from current genome-wide association data [48] or using multiple polygenic scores [49, 50]. However, a high priority for research must be to foster bigger and better genome-wide association studies that can create more powerful polygenic scores. These studies require enormous efforts because samples of unprecedented size are needed to pan for specks of gold from the sand of millions of SNPs. Denigrating polygenic scores because they are ‘only’ predictive undermines this effort.
